# 635. Is Azole Prophylaxis in Liver Transplant Recipients Effective in Preventing Invasive Candidiasis in the Era of Emerging Resistance?

**DOI:** 10.1093/ofid/ofac492.687

**Published:** 2022-12-15

**Authors:** Pooja R Gurram, Fernanda P Silveira, Eun Jeong Kwak, Cornelius Clancy, Minh-Hong Nguyen

**Affiliations:** University of Florida, Gainesville, Florida; University of Pittsburgh Medical Center, Pittsburgh, Pennsylvania; University of Pittsburgh Medical Center, Pittsburgh, Pennsylvania; University of Pittsburgh Medical Center, Pittsburgh, Pennsylvania; University of Pittsburgh, Pittsburgh, PA

## Abstract

**Background:**

Antifungal prophylaxis (px) in liver transplant (LT) recipients reduces invasive candidiasis (IC) and its associated mortality. Studies from our institution (UPMC) and others have shown that targeted px was as effective as universal px. Antifungal resistance has emerged among *Candida* spp., with ∼7% of the bloodstream isolates tested at CDC resistant to fluconazole. We sought to determine if our targeted antifungal px in LT recipients remained effective in the era of rising azole resistance.

**Methods:**

We performed a retrospective study of patients undergoing LT at UPMC between 11/2010 and 06/2021. IC episodes were included if they occurred within 6 months of transplant. IC was defined using EORTC/MSGERC criteria, 2020. CLSI’s M60 2nd edition was used for antifungal susceptibility interpretation. Px was with fluconazole or voriconazole, based on risk factors for yeast or mold infection, respectively (Fig. 1).

**Figure:**
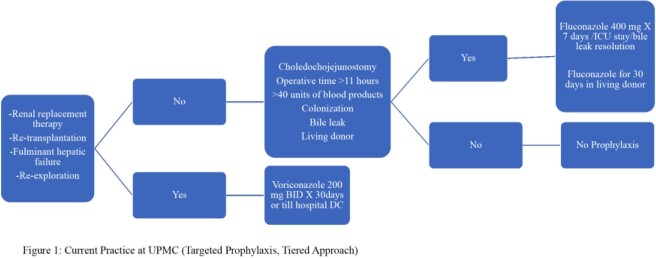

**Results:**

1065 patients (pts) underwent LT over the study period. The most common indications for LT were HCC and NASH cirrhosis. Thirty-four pts (3%) developed IC within six months. 74% and 65% of these pts had risk factors for yeast or mold infection at some point, respectively (Table 1). Twenty-five of 34 (73.5%) pts received antifungal px, with voriconazole most commonly. Sixteen pts with IC (47%) had living donor transplant, and 11 (32%) had Roux-en-Y anastomosis. Types of IC were intra-abdominal candidiasis in 19 (56%), fungemia in 11 (32%), and intra-abdominal IC with secondary fungemia in 4 (12%). Seventeen (50%) episodes were breakthrough (BT) IC (most BT occurred in the voriconazole group), eight (24%) developed after stopping px, and nine (26%) occurred in those without px (Table 2). *C. albicans* and *C. glabrata* were the most common species (Fig. 2). Sixteen isolates underwent susceptibility testing: 12% and 44% were resistant and S-DD to fluconazole (Fig. 2); none were resistant to voriconazole. Attributable mortality of IC was 8%.

**Figure:**
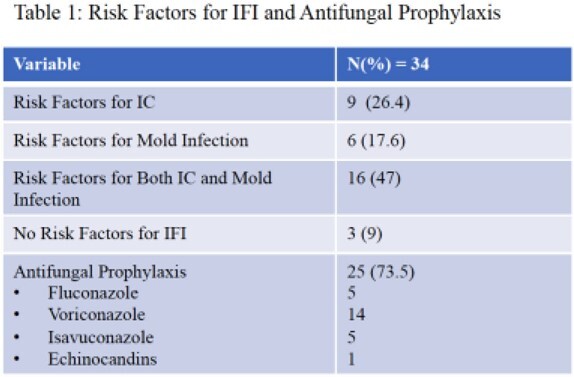

**Figure:**
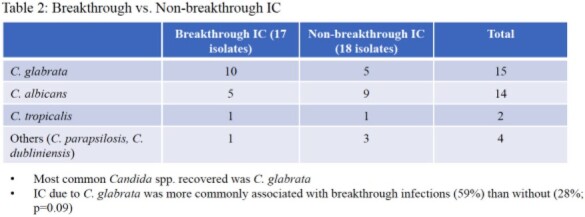

**Figure:**
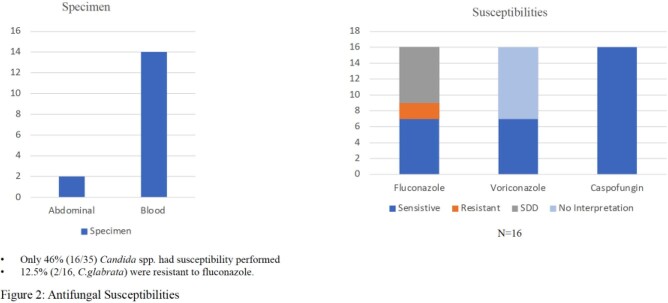

**Conclusion:**

Targeted antifungal px in LT recipients with fluconazole or voriconazole effectively prevented IC, even in the era of rising azole resistance among *Candida* spp. We recommend antifungal susceptibility testing for *Candida* spp. obtained from infected sites to guide antifungal therapy.

**Disclosures:**

**Fernanda P. Silveira, MD**, Ansun: Grant/Research Support|Janssen: Honoraria|Merck: Grant/Research Support|Novartis: Grant/Research Support|Regeneron: Grant/Research Support|Takeda: Advisor/Consultant|Takeda: Grant/Research Support.

